# A novel control of human keratin expression: cannabinoid receptor 1-mediated signaling down-regulates the expression of keratins K6 and K16 in human keratinocytes *in vitro* and *in situ*

**DOI:** 10.7717/peerj.40

**Published:** 2013-02-19

**Authors:** Yuval Ramot, Koji Sugawara, Nóra Zákány, Balázs I. Tóth, Tamás Bíró, Ralf Paus

**Affiliations:** 1 Department of Dermatology, University of Luebeck, Luebeck, Germany; 2 Department of Dermatology, Hadassah-Hebrew University Medical Center, Jerusalem, Israel; 3 Department of Dermatology, Osaka City University Graduate School of Medicine, Osaka, Japan; 4 DE-MTA “Lendület” Cellular Physiology Research Group, Department of Physiology, MHSC, RCMM, University of Debrecen, Debrecen, Hungary; 5 Laboratory of Ion Channel Research and TRP Research Platform Leuven (TRPLe), Department of Cellular and Molecular Medicine, KU Leuven, Leuven, Belgium; 6 Institute of Inflammation and Repair, and Dermatology Centre, University of Manchester, Manchester, UK

**Keywords:** Cannabinoid, Keratin, Psoriasis, Wound healing

## Abstract

Cannabinoid receptors (CB) are expressed throughout human skin epithelium. CB1 activation inhibits human hair growth and decreases proliferation of epidermal keratinocytes. Since psoriasis is a chronic hyperproliferative, inflammatory skin disease, it is conceivable that the therapeutic modulation of CB signaling, which can inhibit both proliferation and inflammation, could win a place in future psoriasis management. Given that psoriasis is characterized by up-regulation of keratins K6 and K16, we have investigated whether CB1 stimulation modulates their expression in human epidermis. Treatment of organ-cultured human skin with the CB1-specific agonist, arachidonoyl-chloro-ethanolamide (ACEA), decreased K6 and K16 staining intensity *in situ*. At the gene and protein levels, ACEA also decreased K6 expression of cultured HaCaT keratinocytes, which show some similarities to psoriatic keratinocytes. These effects were partly antagonized by the CB1-specific antagonist, AM251. While CB1-mediated signaling also significantly inhibited human epidermal keratinocyte proliferation *in situ*, as shown by K6/Ki-67-double immunofluorescence, the inhibitory effect of ACEA on K6 expression *in situ* was independent of its anti-proliferative effect. Given recent appreciation of the role of K6 as a functionally important protein that regulates epithelial wound healing in mice, it is conceivable that the novel CB1-mediated regulation of keratin 6/16 revealed here also is relevant to wound healing. Taken together, our results suggest that cannabinoids and their receptors constitute a novel, clinically relevant control element of human K6 and K16 expression.

## Introduction

Endocannabinoids as well as exocannabinoids (such as the active components of cannabis) control the function of various types of cells *via* cannabinoid receptor (CB)-dependent or independent manner (Kupczyk, Reich & Szepietowski, 2009). The endocannabinoid system (ECS) consists of these CBs, their endogenous ligands (i.e. endocannabinoids, such as anandamide [AEA] and 2-arachidonoylglycerol), and enzymes responsible for endocannabinoid synthesis and degradation (Biro et al., 2009). In human skin, many different types of cells are now known to express functional CBs (Biro et al., 2009; Czifra et al., 2012; Pucci et al., 2012; Roelandt et al., 2012; Stander et al., 2005; Sugawara et al., 2012; Telek et al., 2007; Toth et al., 2011). The ECS is increasingly appreciated as an important regulator of skin function in health and disease. For example, the ECS has become implicated in pain (Khasabova et al., 2012; Walker & Hohmann, 2005) and itch control (Stander, Reinhardt & Luger, 2006), and the modulation of inflammation (Klein, 2005) and allergy (Karsak et al., 2007). In addition, CB1 signaling is important in mast cell activation and intracutaneous mast cell maturation from resident progenitors (Sugawara et al., 2012). Furthermore, it regulates fibrosis (Akhmetshina et al., 2009), sebocyte differentiation (Dobrosi et al., 2008) and eccrine epithelial biology (Czifra et al., 2012). Nevertheless, the functions of CB-mediated signaling in human keratinocytes (KCs) *in situ* are as yet poorly understood.

We have previously shown that outer root sheath KCs of human hair follicles (HFs) express CB1. CB1 stimulation by the endocannabinoid, AEA, markedly inhibited human HF growth by inhibiting hair matrix KC proliferation and inducing apoptosis, thus leading to premature HF involution (catagen development). This was reversed by the CB1-specific antagonist, AM251 (Telek et al., 2007). Similarly, human epidermal KC express CBs, and their differentiation is regulated *via* CB1 (Maccarrone et al., 2003; Paradisi et al., 2008). AEA also markedly suppresses human epidermal KC proliferation and induces apoptosis *via* CB1 *in vitro* and *in situ* (Toth et al., 2011). This suggests that the ECS could become a useful therapeutic target in the management of chronic hyperproliferative human skin diseases, such as psoriasis (Toth et al., 2011).

However, it remains unclear whether and how CB1-mediated signaling impacts on human KC differentiation, namely on the expression of hyperproliferation-associated keratins. Psoriasis is characterized by the upregulation of keratins K6 and K16 expression within lesional epidermis (Korver et al., 2006; Mommers et al., 2000). This pair of keratins is also prominently up-regulated in the epidermis under wound healing conditions in men and mice (Moll, Divo & Langbein, 2008; Paladini et al., 1996; Rotty & Coulombe, 2012) and is constitutively expressed in the outer root sheath KCs of human HFs (Langbein & Schweizer, 2005; Moll, Divo & Langbein, 2008; Ramot et al., 2009). Psoriasis is a chronic inflammatory, hyperproliferative dermatosis that, in addition to its anti-proliferative properties (Telek et al., 2007; Van Dross et al., 2012), might also profit from the well-recognized anti-inflammatory properties of CB1-mediated signaling (Sugawara et al., 2012; Wilkinson & Williamson, 2007). Therefore, we have investigated whether CB1 stimulation modulates K6 and K16 expression in human skin. This question was made particularly interesting in view of the most recent discovery that, in murine skin, K6 is not only a wound healing-associated keratin, but actively down-regulates KC migration during wound repair (Rotty & Coulombe, 2012).

In order to answer this question, we used the CB1-specific agonist, arachidonoyl-chloro-ethanolamide (ACEA) (Harvey et al., 2012), and checked its effect on K6 expression *in situ*. This was done by utilizing full thickness human skin organ culture (Lu et al., 2007) as a physiologically and clinically relevant model to study multiple aspects of human skin biology under clinically relevant conditions *in vitro* (Bodo et al., 2010; Knuever et al., 2012; Langan et al., 2010; Lu et al., 2007; Sugawara et al., 2012). In order to confirm the CB1-specificity of the observed effects of ACEA, we also used the CB1-specific antagonist, AM251 (Chanda et al., 2011).

K16 serves as the type I keratin partner of K6 in the formation of intermediate filament heterodimers (Moll, Divo & Langbein, 2008). It is also involved in epidermal barrier function (Grzanka et al., 2012; Thakoersing et al., 2012), and is up-regulated in hyperprolifeative conditions of the skin such as psoriasis (Iizuka et al., 2004) and atopic dermatitis (Grzanka et al., 2012). Therefore, we also examined the effects of ACEA on K16 expression.

HaCaT cells are a highly proliferating human KC line known to overexpress K6 (Ryle et al., 1989). Since HaCaT KCs share some other characteristics with psoriatic KCs and are often employed as surrogate “psoriatic” KCs (Balato et al., 2012; Farkas et al., 2001; George et al., 2010; Kim et al., 2011; Ronpirin & Tencomnao, 2012; Saelee, Thongrakard & Tencomnao, 2011), we also tested whether and how ACEA modulated K6 expression in these cells *in vitro*. In order to delineate whether any such effects on keratin expression resulted only indirectly from a possible down-regulation of KC proliferation by CB1 stimulation (Toth et al., 2011), double-labeling and quantitative immunohistomorphometry for both K6 and Ki-67 was performed. Finally, to investigate whether K6-expressing human KCs co-express CB1, double-immunolabeling for both antigens was employed.

## Materials and methods

### Human skin organ culture

Isolated human skin samples obtained from elective plastic surgery procedures (32 pieces of skin fragments obtained by 4 mm punch biopsies from 4 individuals; 3 females and a male aged 26–74, average: 56.5; 3 skin samples were taken from the scalp and one was taken from the hip) were maintained in supplemented serum-free William’s E medium as previously reported (Bodo et al., 2010; Knuever et al., 2012; Lu et al., 2007; Poeggeler et al., 2010). Human tissue collection and handling was performed according to Helsinki guidelines, after institutional review board ethics approval (University of Luebeck) and informed patient consent.

Skin samples were first incubated overnight to adapt to culture conditions after which the medium was replaced and vehicle or test substances were added. For human skin organ culture, skin samples were treated with ACEA (Sigma-Aldrich, Taufkirchen, Germany, 30 µM) or AM251 (Sigma-Aldrich, 1 µM), or the combination of them for 1-day after the overnight incubation (Sugawara et al., 2012). Following culturing for the time indicated, skin samples were cryoembedded and prepared for histology, immunohistochemistry/immunofluorescence and quantitative immunohistomorphometry (Ramot et al., 2010; Ramot et al., 2011; Sugawara et al., 2012). Each evaluation was performed on 2–4 sections of 2 skin fragments per each treatment group from 2–4 individuals.

### Cell culture

Human immortalized HaCaT KCs (Boukamp et al., 1988) were cultured in DMEM (Sigma-Aldrich) supplemented with 10% fetal bovine serum (Invitrogen, Paisley, UK) and antibiotics (PAA Laboratories, Pasching, Austria). For qRT-PCR, the cells were cultured with ACEA (1 µM) for 8 h.

### qRT-PCR

qRT-PCR was performed on an ABI Prism 7000 sequence detection system (Applied Biosystems/Life Technologies, Foster City, CA, USA) using the 5’ nuclease assay as detailed in our previous reports (Toth et al., 2011; Toth et al., 2009). Total RNA was isolated from HaCaT keratinocytes using TRIreagent (Applied Biosystems/Life Technologies, Foster City, CA, USA) and digested with recombinant RNase-free DNase-1 (Applied Biosystems) according to the manufacturer’s protocol. After isolation, one µg of total RNA was reverse-transcribed into cDNA by using High Capacity cDNA kit (Applied Biosystems) following the manufacturer’s protocol. PCR amplification was performed by using specific TaqMan primer and probes (Applied Biosystems, assay ID: Hs01699178_g1 for human K6A). As internal housekeeping gene control, transcripts of cyclophillin A (PPIA) were determined (Assay ID: Hs99999904 for human PPIA). The amount of the K6A transcripts was normalized to the control gene using the ΔCT method.

### Immunohistochemistry

For the detection of K6 in organ cultured human skin as well as cultured HaCaT KCs, indirect immunofluorescence staining was performed using mouse anti-human K6 antibody (Progen, Ks6.KA12) at 1:10 dilution as a primary antibody and rhodamine conjugated goat anti-mouse IgG (Jackson Immunoresearch Laboratories, West Grove, PA) at 1:200 dilution in phosphate-buffered saline (PBS) as a secondary antibody.

To study the proliferation of epidermal KCs, double-immunostaining for K6 and Ki-67 was performed. Briefly, after the staining for K6 with FITC conjugated goat anti-mouse IgG (Jackson Immunoresearch Laboratories) as a secondary antibody, sections were incubated overnight at 4 °C with a mouse anti-human Ki-67 antibody (DAKO, Hamburg, Germany) at 1:20 in PBS. Sections were then washed with PBS, followed by incubation with rhodamine conjugated goat anti-mouse IgG (Jackson Immunoresearch Laboratories) (1:200 in PBS, 45 min) at room temperature.

To investigate the localization of CB1 and K6, double immunostaining was performed. For CB1 immunostaining, the highly sensitive tyramide signal amplification (TSA) technique (Perkin Elmer, Boston, MA) was applied. Cryosections were incubated overnight at 4 °C with rabbit anti-CB1 (Santa Cruz, CA, USA) at 1:400 diluted in TNB (Tris, NaOH, Blocking reagent, TSA kit; Perkin-Elmer). Thereafter, the samples were labeled with goat biotinylated antibody against rabbit IgG (Jackson Immunoresearch Laboratories) at 1:200 in TNB for 45 min at room temperature. Sections were then stained with streptavidin-conjugated horseradish peroxidase (1:100, 30 min, TSA kit) and were finally incubated with rhodamine conjugated tyramide (1:50, TSA kit). The TSA method was applied according to the manufacturer’s protocol. For the second primary labeling, mouse anti-human K6 antibody (Progen) was applied at 1:20 in PBS, overnight at 4 °C. After the wash with PBS, the sections were incubated with FITC conjugated goat anti-mouse IgG (Jackson Immunoresearch Laboratories) (1:200 in PBS, 45 min) at room temperature.

For K16 antigen detection, we used the LSAB (DCS, Germany) detection method (Ramot et al., 2009) using the K16-gp as primary antibody (guinea-pig, PROGEN, Heidelberg, Germany, dilution 1:1000, GP-CK16), and biotinylated goat anti-guinea pig as secondary antibody (Vector Laboratories, Burlingame, CA, USA, dilution 1:200).  HistoGreen (Linaris, Wertheim-Bettingen, Germany) was used as peroxidase substrate.

For all immunostainings, the respective primary antibodies were omitted as negative controls, and morphological criteria and reproduction of the previously published intracutaneous expression patterns of the examined antigens were used as internal positive and negative controls (Moll, Divo & Langbein, 2008). For all experiments, control and treated sections were stained (and later evaluated) on the same day by the same investigator. To avoid staining biases, we calculated the relative staining intensity (arbitrary intensity; 1 as control group) among treatment groups per each individual and then pooled data from all of the experiments.

High magnification images of K6/Ki67 double immunofluorescence and K6 immunofluorescence on HaCaT cells were taken by laser scanning confocal microscopy (Fluoview 300, Olympus Tokyo, Japan) running Fluoview 2.1 software (Olympus).

The staining intensity of K6 and K16 in defined reference areas was assessed by quantitative immunohistomorphometry using the ImageJ software (NIH: National Institutes of Health, Bethesda, MD) (Bodo et al., 2010; Ramot et al., 2010; Ramot et al., 2011). For epidermal evaluation, staining intensity was evaluated in the suprabasal cells, and for HaCaT cells, staining intensity was measured in the colonies formed. For K6 immunofluorescence intensity, skin sections from 4 different individuals were used, while K16 immunofluorescence intensity and %Ki67 were evaluated in skin sections from 2 patients. 4–6 sections per one individual (2 sections per investigated skin fragment) were used for each evaluation. Each section was evaluated in two/three different non-adjacent microscopic fields (×200), and the mean intensity was measured, and considered as a value. Each treatment group was compared to the control group (average value), and relative change in expression was calculated. Highly comparable results were obtained from different sections from different individuals.

## Statistical analysis

Significance of difference between two groups was evaluated using Student’s t-test for unpaired samples. For multiple comparisons, one-way analysis of variance (ANOVA) was used, followed by Bonferroni’s multiple comparison test, using Prism 5.0 software (GraphPad Prism Program, GraphPad, San Diego, CA). *p* values <0.05 were regarded as significant. All data in the figures are expressed as mean + SEM. *p < 0.05, **p < 0.01, ***p < 0.001 for the indicated comparisons.

## Results and discussion

### The CB1-selective agonist, ACEA, down-regulates K6 protein expression *in situ*

**Figure 1:**
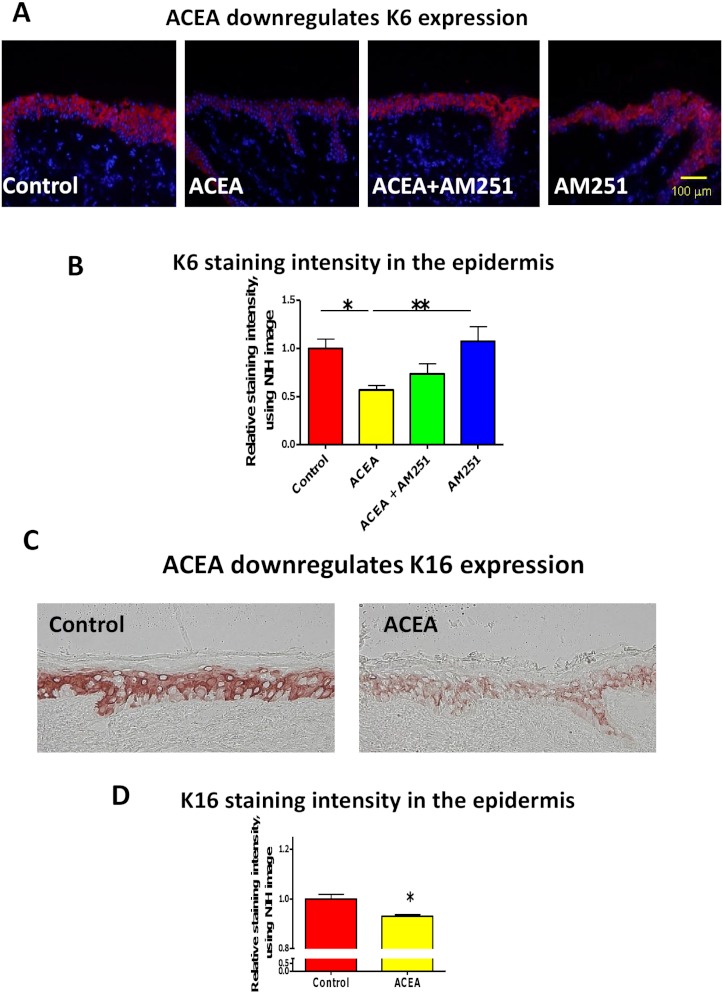
The CB1 specific agonist, ACEA significantly inhibits K6 and K16 expression *in situ*. (A) Representative images of K6 immunofluorescence with organ cultured human skin treated with ACEA/AM251 (1-day). (B) Statistical analysis of K6 immunofluorescence intensity in organ cultured human skin (quantitative immunohistomorphemtry, ImageJ); stimulation with ACEA (30 µM), AM251 (1 µM) or both for 1-day. *n* = 9–22 skin sections/group. (C) Representative images of K16 immunohistochemistry with organ cultured human skin samples with ACEA (1-day). (D) Quantitative K16 immunohistomorphometry within the epidermis of organ-cultured human skin samples after 1-day of stimulation with ACEA (30 µM). *n* = 4 skin sections/group. Data are expressed as mean + SEM. *p < 0.05, **p < 0.01.

First, we asked whether the CB1-specific synthetic agonist ACEA (Pertwee et al., 2010; Sugawara et al., 2012) can modulate the expression of keratin K6 in human skin. K6 staining intensity within the epidermis of full-thickness human skin that had been organ-cultured for 24 h under serum-free conditions in the presence of ACEA (30 µM) or vehicle alone was assessed by quantitative immunohistomorphometry. This showed that K6 immunoreactivity (IR) was significantly reduced after ACEA treatment, compared to the vehicle control group (Figs. 1A and 1B).

This down-regulation was abrogated in part by the co-administration with the CB1-specific antagonist, AM251 (Pertwee et al., 2010; Sugawara et al., 2012) (Figs. 1A and 1B). Therefore, intraepidermal K6 protein expression in normal human skin *in situ* is down-regulated in a CB1-specific manner.

### ACEA also down-regulates K16 protein expression *in situ*

Since K16 is the type I keratin partner of K6 in KCs and is thought to stabilize this keratin protein as a cytoskeletal heteropolymeric intermediate filament (Ramot et al., 2009), we next analyzed K16 IR in the epidermis of organ cultured human skin samples treated with ACEA. In line with the K6 protein expression, K16 IR was also significantly down-regulated by ACEA *in situ* (Figs. 1C and 1D).

### CB1-mediated signaling also regulates K6 expression in cultured, hyperproliferative human keratinocytes

In order to check whether the observed CB1-mediated effects on K6 regulation within intact human skin epithelium depend on intact epithelial-mesenchymal interactions between epidermis and dermis, or are likely to reflect a direct impact of CB1 ligands on epidermal keratinocytes, we next investigated K6 expression in cultured human HaCaT KCs. This transformed human KC line is well-appreciated to constitutively express K6 and to be hyperproliferative (just like human wounded and psoriatic KC) (Balato et al., 2012; Farkas et al., 2001; George et al., 2010; Kim et al., 2011; Ronpirin & Tencomnao, 2012; Ryle et al., 1989; Saelee, Thongrakard & Tencomnao, 2011). K6 is expressed in hyper-proliferative cells (Weiss, Eichner & Sun, 1984) and both K6 expression and basal layer epidermal KC proliferation are increased in psoriasis lesions (Donetti et al., 2012; Griffiths & Barker, 2007; Korver et al., 2006; Litvinov et al., 2011; Mommers et al., 2000). HaCaT cells are known to express functional CB1 and CB2 (Leonti et al., 2010; Maccarrone et al., 2003; Paradisi et al., 2008), and this had been confirmed previously by our group, both on the gene (RT-PCR analysis) and protein levels (immunocytochemistry and western blotting techniques) (Toth et al., 2011). Thus, being a direct target of CB1-mediated signaling, this makes these KCs not only an instructive cell culture tool for evaluating the direct, dermis-independent role of CB1-mediated signaling in the regulation of keratin expression in human KCs, but may also provide first indications as to how the observed K6 expression could relate to wound healing and/or psoriasis.

**Figure 2:**
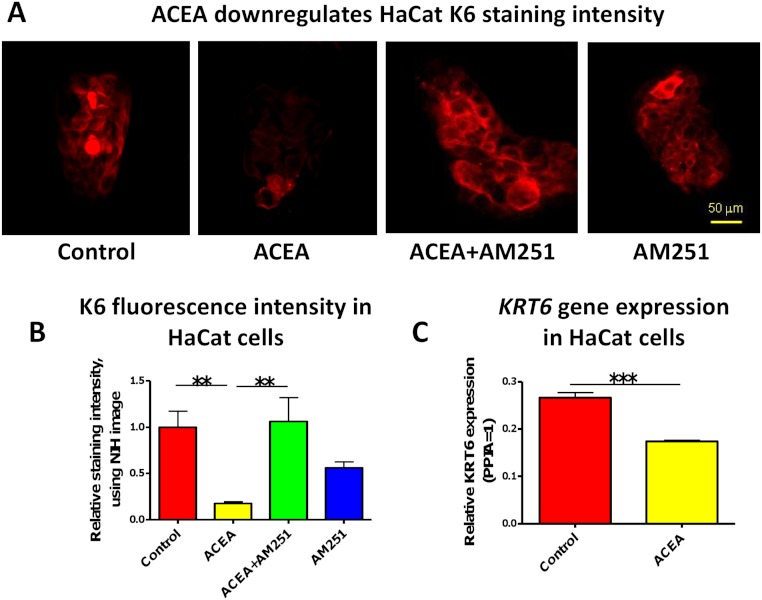
The CB1 specific agonist, ACEA significantly inhibits K6 expression in cultured HaCaT cells. (A) Representative images of K6 immunofluorescence of cultured HaCaT KCs with ACEA (1 µM), AM251 (100 nM) or both for 1-day. (B) Statistical analysis of K6 immunofluorescence intensity of cultured HaCaT cells. *n* = 6 colonies/group (C) Statistical analysis of K6 gene expression in HaCaT cells treated with vehicle control or ACEA (1 µM) for 8 h. Data are expressed as mean + SEM. *p < 0.05; **p < 0.01; ***p < 0.001.

In accordance with our human skin organ culture results, ACEA (1 µM) significantly down-regulated K6 staining intensity of HaCaT cells *in vitro* (Figs. 2A and 2B). This was abrogated by the co-administration of the selective CB1 antagonist, AM251 (100 nM) (Figs. 2A and 2B). Unexpectedly, though, AM251 alone had a partial inhibitory effect on K6 expression, although not significant. This may be related to the fact that AM251 is an inverse agonist (Dono & Currie, 2012; Fiori et al., 2011), and invites further study. The inhibitory effect of ACEA on K6 protein expression was further confirmed by quantitative RT-PCR (Fig. 2C). Therefore, while HaCaT cells may exhibit relatively low CB expression levels, at least under our assay conditions, they showed a vigorous response to CB ligands.

### The CB1 specific agonist, ACEA significantly decreases human epidermal keratinocyte proliferation *in situ*

However, the observed effects of CB1-mediated signaling on epidermal K6 expression could simply reflect the appreciated anti-proliferative effects of CB1 agonists (Casanova et al., 2003; Hermanson & Marnett, 2011; Toth et al., 2011). Moreover, K6 is overexpressed in hyper-proliferative and wounded KCs (Weiss, Eichner & Sun, 1984), and both K6 expression and basal KC proliferation are increased in psoriatic epidermal lesions (Donetti et al., 2012; Griffiths & Barker, 2007; Korver et al., 2006; Litvinov et al., 2011; Mommers et al., 2000; Navarro, Casatorres & Jorcano, 1995). Therefore, we next assessed whether CB1 stimulation by CB1 specific agonist, ACEA, could affect human KC proliferation *in situ*.

**Figure 3:**
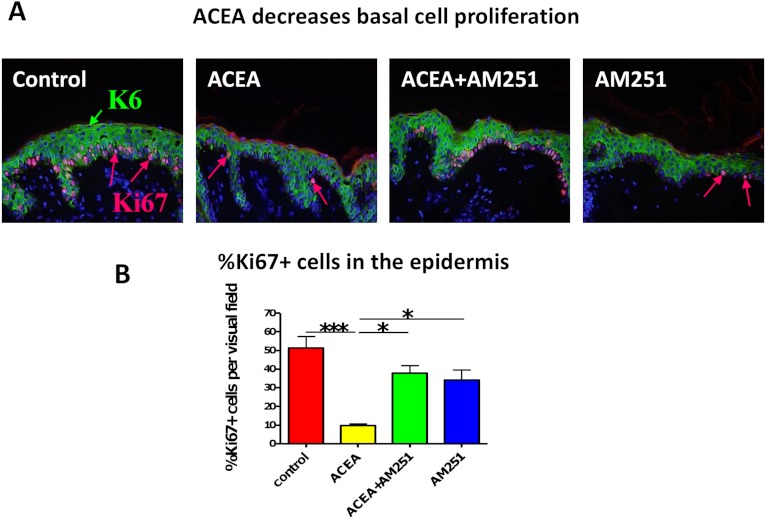
The CB1 specific agonist, ACEA significantly decreases human epidermal keratinocyte proliferation *in situ.* (A) Representative images of K6 (green) and Ki67 (red) double-immunofluorescence with organ cultured human skin treated with ACEA/AM251 (1-day). (B) Quantitative analysis of the percentage of Ki67 + KCs within organ cultured human epidermis. *p < 0.05; ***p < 0.001. *n* = 5–12 skin sections/group.

Just as we had seen before with the non-selective endocannabinoid, AEA (Toth et al., 2011), the CB1-specific synthetic agonist, ACEA indeed significantly decreased human epidermal KC proliferation *in situ*. This effect was abrogated by the CB1-specific antagonist, AM251 (assessed by quantitative Ki-67 immunomorphometry, Figs. 3A and 3B).

### The CB1 specific agonist, ACEA, significantly decreases K6 expression in suprabasal cells in a proliferation-independent manner

**Figure 4:**
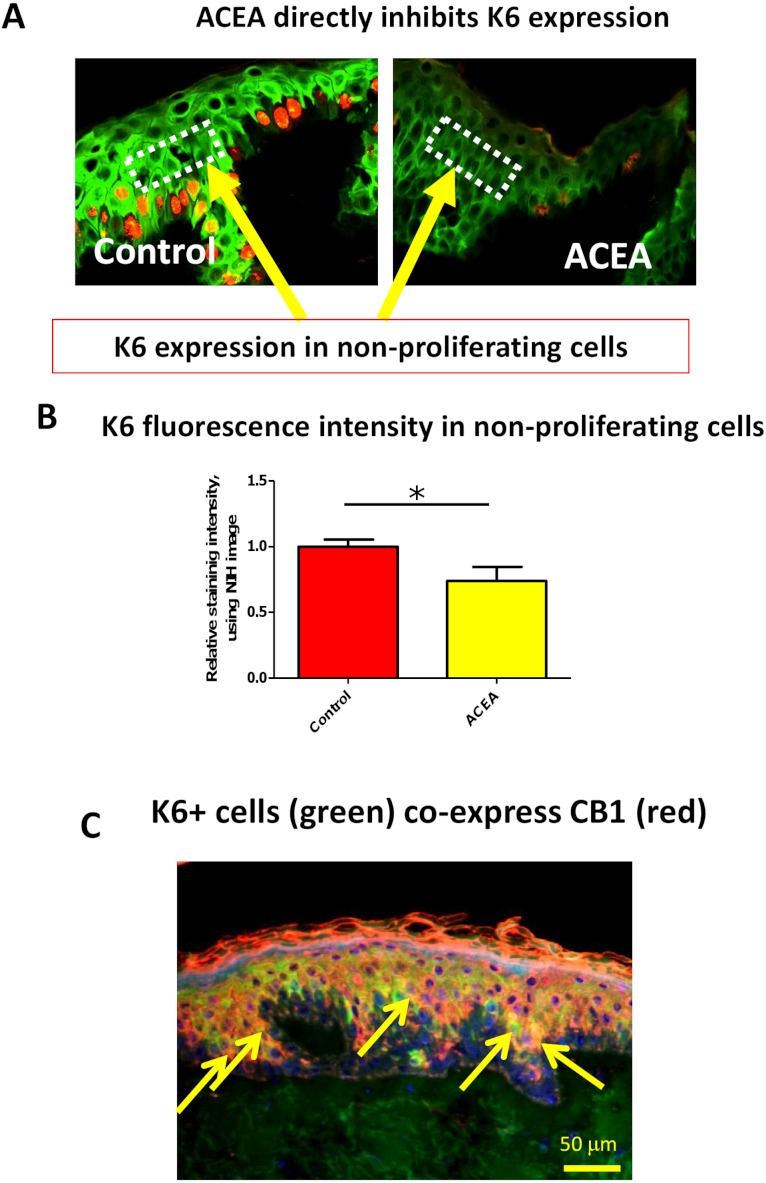
The CB1 specific agonist, ACEA, significantly decreases K6 expression in suprabasal cells in a proliferation-independent manner. (A) Representative images of K6 (green) and Ki-67 (red) double immunofluorescence. Dotted rectangles indicate the reference area for quantitative immunohistomorphometry of K6 fluorescence intensity. (B) Quantitative analysis of K6 fluorescence intensity in non-proliferating (i.e. Ki67-negative) cells within human epidermis *in situ*. Data are expressed as mean + SEM. *p < 0.05. *n* = 5–7 skin sections/group. (C) K6 (green) and CB1 (red) double-immunofluorescence study. Yellow arrows denote double-positive KCs.

Therefore, it needed to be dissected whether or not CB1 also reduces K6 expression in a proliferation-independent manner. This was done by selectively assessing K6 expression in non-proliferative (i.e. Ki-67-negative) epidermal KCs *in situ*. We found that K6 IR within non-proliferative epidermis was also reduced by ACEA (Figs. 4A and 4B). Furthermore, K6-expressing cells in the epidermis co-expressed CB1 *in situ* (Fig. 4C), suggesting a direct effect of CB1-agnosits on K6-expressing human epidermal KCs *in situ*. Thus, CB1 stimulation may affect K6 expression both, by reducing KC proliferation and by down-regulating K6 expression directly *via* CB1 in a proliferation-independent manner.

Here we provide the first evidence that CB1-mediated signaling directly regulates K6/16 expression within normal human skin. Specifically, we show that CB1 stimulation down-regulates expression of the hyper-proliferation-associated human keratins K6 *in vitro* and *in situ*, and inhibits human epidermal KC proliferation *in situ*.

The effect of CB-mediated signaling in human KC biology remains to be fully explored. As we have also observed in isolated human skin (Fig. 4C), CB1 protein expression is detected mainly above the basal layer of the epidermis (Stander et al., 2005), i.e. above the compartment where KC proliferation normally occurs most prominently. Wilkinson and Williamson reported that the non-selective CB agonist HU210 inhibited KC proliferation. However, this could not be blocked by either CB1 or CB2 antagonists, suggesting that cannabinoids may also inhibit human KC proliferation through a non-CB1/CB2 mechanism (Wilkinson & Williamson, 2007). Nevertheless, it has been previously shown that AEA, which can interact with CB1 on human KC (Biro et al., 2009), inhibited human KC proliferation *in situ* and *in vitro* (Toth et al., 2011).

Therefore, it was important to clarify whether specific CB1 stimulation inhibits human epidermal KC proliferation *in situ*. By using CB1-specific agonists and antagonists we clearly demonstrate that exclusive CB1 stimulation inhibited KC proliferation. Thus, CB1 is an important key regulator of human KC proliferation. Given the role of epidermal hyperproliferation in the pathobiology of psoriasis (Donetti et al., 2012; Griffiths & Barker, 2007; Korver et al., 2006; Litvinov et al., 2011; Mommers et al., 2000; Navarro, Casatorres & Jorcano, 1995), cannabimimetic agents that activate CB1, therefore, deserve consideration as a novel pharmacological strategy for treating psoriasis.

Furthermore, increased numbers of activated mast cells are often observed in and around lesional psoriatic skin, and increasing evidence suggests that mast cells are functionally important key immunocytes in the pathogenesis of psoriasis (Carvalho, Nilsson & Harvima, 2010; Namazi, 2005; Radosa et al., 2011; Suttle et al., 2012; Toruniowa & Jablonska, 1988). Recently, we have shown that CB1 activation limits excessive mast cell activity and even inhibits mast cell maturation of resident, intracutaneous progenitors (Sugawara et al., 2012). Therefore, besides their anti-proliferative effects on human epidermal KCs, the anti-inflammatory (Richardson, Kilo & Hargreaves, 1998) and mast cell-inhibitory properties of CB1 agonists in human skin (Sugawara et al., 2012) make them a particularly attractive class of agents in future psoriasis management.

It should be noted, that the constitutive level of K6 expression in organ-cultured human skin fragments is considerably higher than normal scalp skin *in vivo*. Presumably, this occurs as a response to tissue dissection and organ culture, which is well-known to elicit an immediate wound healing response in the epithelium. The latter rapidly starts to migrate over the wound edge in an attempt to enwrap the exposed skin mesenchyme (epiboly phenomenon Stenn, 1981; Brown et al., 1991). This constitutive up-regulation of K6 in organ-cultured normal human skin may greatly heighten the sensitivity to K6 expression-modulatory compounds, such as CB ligands, thus making human skin organ culture a particularly sensitive and instructive tool for clinically relevant keratin research. At the same time, however, caution is advised in extrapolating from these observation made in what essentially reflects a wound healing milieu to healthy, unmanipulated human skin *in vivo*.

The current findings invite the speculation that the therapeutic down-modulation of K6 and/or K16 expression by CB1 agonists and other cannabimimetics might become exploitable for the management of other dermatoses besides psoriasis, for example pachyonychia congenita (Hickerson et al., 2011; Zhao et al., 2011) and acne (Biro et al., 2009), and could be used to modulate KC migration-dependent reepithelialization in wound healing, similar to related findings in periodontal and intestinal wound repair (Kozono et al., 2010; Wright et al., 2005).

## Conclusion

Our results suggest that cannabinoids and their receptors constitute a novel, clinically relevant control element of human K6 and K16 expression. Therefore, cannabimimetic agents might be relevant for the treatment of several skin conditions related to aberrant K6/K16 expression, such as psoriasis and wound healing. In addition, skin organ culture is shown to be a clinically and physiologically relevant model system for investigating the effect of CB1 specific agonists/antagonists on human skin.

**Abbreviations**
ACEA: arachidonoyl-chloro-ethanolamide
AEA: anandamide
CB1: cannabinoid receptor 1
ECS: endocannabinoid system
HF: hair follicle
KC: keratinocyte

## References

[ref-1] Akhmetshina A, Dees C, Busch N, Beer J, Sarter K, Zwerina J, Zimmer A, Distler O, Schett G, Distler JH (2009). The cannabinoid receptor CB2 exerts antifibrotic effects in experimental dermal fibrosis. Arthritis and Rheumatism.

[ref-2] Balato A, Lembo S, Mattii M, Schiattarella M, Marino R, De Paulis A, Balato N, Ayala F (2012). IL-33 is secreted by psoriatic keratinocytes and induces pro-inflammatory cytokines via keratinocyte and mast cell activation. Experimental Dermatology.

[ref-3] Biro T, Toth BI, Hasko G, Paus R, Pacher P (2009). The endocannabinoid system of the skin in health and disease: novel perspectives and therapeutic opportunities. Trends in Pharmacological Sciences.

[ref-4] Bodo E, Kany B, Gaspar E, Knuver J, Kromminga A, Ramot Y, Biro T, Tiede S, van Beek N, Poeggeler B, Meyer KC, Wenzel BE, Paus R (2010). Thyroid-stimulating hormone, a novel, locally produced modulator of human epidermal functions, is regulated by thyrotropin-releasing hormone and thyroid hormones. Endocrinology.

[ref-5] Boukamp P, Petrussevska RT, Breitkreutz D, Hornung J, Markham A, Fusenig NE (1988). Normal keratinization in a spontaneously immortalized aneuploid human keratinocyte cell line. Journal of Cell Biology.

[ref-6] Brown C, Stenn KS, Falk RJ, Woodley DT, O’Keefe EJ (1991). Vitronectin: effects on keratinocyte motility and inhibition of collagen-induced motility. Journal of Investigative Dermatology.

[ref-7] Carvalho RF, Nilsson G, Harvima IT (2010). Increased mast cell expression of PAR-2 in skin inflammatory diseases and release of IL-8 upon PAR-2 activation. Experimental Dermatology.

[ref-8] Casanova ML, Blazquez C, Martinez-Palacio J, Villanueva C, Fernandez-Acenero MJ, Huffman JW, Jorcano JL, Guzman M (2003). Inhibition of skin tumor growth and angiogenesis in vivo by activation of cannabinoid receptors. Journal of Clinical Investigation.

[ref-9] Chanda D, Kim DK, Li T, Kim YH, Koo SH, Lee CH, Chiang JY, Choi HS (2011). Cannabinoid receptor type 1 (CB1R) signaling regulates hepatic gluconeogenesis via induction of endoplasmic reticulum-bound transcription factor cAMP-responsive element-binding protein H (CREBH) in primary hepatocytes. Journal of Biological Chemistry.

[ref-10] Czifra G, Szollosi AG, Toth BI, Demaude J, Bouez C, Breton L, Biro T (2012). Endocannabinoids regulate growth and survival of human eccrine sweat gland-derived epithelial cells. Journal of Investigative Dermatology.

[ref-11] Dobrosi N, Toth BI, Nagy G, Dozsa A, Geczy T, Nagy L, Zouboulis CC, Paus R, Kovacs L, Biro T (2008). Endocannabinoids enhance lipid synthesis and apoptosis of human sebocytes via cannabinoid receptor-2-mediated signaling. FASEB Journal.

[ref-12] Donetti E, Gualerzi A, Ricceri F, Pescitelli L, Bedoni M, Prignano F (2012). Etanercept restores a differentiated keratinocyte phenotype in psoriatic human skin: a morphological study. Experimental Dermatology.

[ref-13] Dono LM, Currie PJ (2012). The cannabinoid receptor CB(1) inverse agonist AM251 potentiates the anxiogenic activity of urocortin I in the basolateral amygdala. Neuropharmacology.

[ref-14] Farkas A, Kemeny L, Szony BJ, Bata-Csorgo Z, Pivarcsi A, Kiss M, Szell M, Koreck A, Dobozy A (2001). Dithranol upregulates IL-10 receptors on the cultured human keratinocyte cell line HaCaT. Inflammation Research.

[ref-15] Fiori JL, Sanghvi M, O’Connell MP, Krzysik-Walker SM, Moaddel R, Bernier M (2011). The cannabinoid receptor inverse agonist AM251 regulates the expression of the EGF receptor and its ligands via destabilization of oestrogen-related receptor alpha protein. British Journal of Pharmacology.

[ref-16] George SE, Anderson RJ, Cunningham A, Donaldson M, Groundwater PW (2010). Evaluation of a range of anti-proliferative assays for the preclinical screening of anti-psoriatic drugs: a comparison of colorimetric and fluorimetric assays with the thymidine incorporation assay. Assay and Drug Development Technologies.

[ref-17] Griffiths CE, Barker JN (2007). Pathogenesis and clinical features of psoriasis. Lancet.

[ref-18] Grzanka A, Zebracka-Gala J, Rachowska R, Bozek A, Kowalska M, Jarzab J (2012). The effect of pimecrolimus on expression of genes associated with skin barrier dysfunction in atopic dermatitis skin lesions. Experimental Dermatology.

[ref-19] Harvey BS, Ohlsson KS, Maag JL, Musgrave IF, Smid SD (2012). Contrasting protective effects of cannabinoids against oxidative stress and amyloid-beta evoked neurotoxicity *in vitro*. Neurotoxicology.

[ref-20] Hermanson DJ, Marnett LJ (2011). Cannabinoids, endocannabinoids, and cancer. Cancer and Metastasis Reviews.

[ref-21] Hickerson RP, Leachman SA, Pho LN, Gonzalez-Gonzalez E, Smith FJ, McLean WH, Contag CH, Leake D, Milstone LM, Kaspar RL (2011). Development of quantitative molecular clinical end points for siRNA clinical trials. Journal of Investigative Dermatology.

[ref-22] Iizuka H, Takahashi H, Honma M, Ishida-Yamamoto A (2004). Unique keratinization process in psoriasis: late differentiation markers are abolished because of the premature cell death. Journal of Dermatology.

[ref-23] Karsak M, Gaffal E, Date R, Wang-Eckhardt L, Rehnelt J, Petrosino S, Starowicz K, Steuder R, Schlicker E, Cravatt B, Mechoulam R, Buettner R, Werner S, Di Marzo V, Tuting T, Zimmer A (2007). Attenuation of allergic contact dermatitis through the endocannabinoid system. Science.

[ref-24] Khasabova IA, Khasabov S, Paz J, Harding-Rose C, Simone DA, Seybold VS (2012). Cannabinoid type-1 receptor reduces pain and neurotoxicity produced by chemotherapy. Journal of Neuroscience.

[ref-25] Kim TG, Byamba D, Wu WH, Lee MG (2011). Statins inhibit chemotactic interaction between CCL20 and CCR6 in vitro: possible relevance to psoriasis treatment. Experimental Dermatology.

[ref-26] Klein TW (2005). Cannabinoid-based drugs as anti-inflammatory therapeutics. Nature Reviews Immunology.

[ref-27] Knuever J, Poeggeler B, Gaspar E, Klinger M, Hellwig-Burgel T, Hardenbicker C, Toth BI, Biro T, Paus R (2012). Thyrotropin-releasing hormone controls mitochondrial biology in human epidermis. Journal of Clinical Endocrinology and Metabolism.

[ref-28] Korver JE, van Duijnhoven MW, Pasch MC, van Erp PE, van de Kerkhof PC (2006). Assessment of epidermal subpopulations and proliferation in healthy skin, symptomless and lesional skin of spreading psoriasis. British Journal of Dermatology.

[ref-29] Kozono S, Matsuyama T, Biwasa KK, Kawahara K, Nakajima Y, Yoshimoto T, Yonamine Y, Kadomatsu H, Tancharoen S, Hashiguchi T, Noguchi K, Maruyama I (2010). Involvement of the endocannabinoid system in periodontal healing. Biochemical and Biophysical Research Communications.

[ref-30] Kupczyk P, Reich A, Szepietowski JC (2009). Cannabinoid system in the skin – a possible target for future therapies in dermatology. Experimental Dermatology.

[ref-31] Langan EA, Ramot Y, Hanning A, Poeggeler B, Biro T, Gaspar E, Funk W, Griffiths CE, Paus R (2010). Thyrotropin-releasing hormone and oestrogen differentially regulate prolactin and prolactin receptor expression in female human skin and hair follicles *in vitro*. British Journal of Dermatology.

[ref-32] Langbein L, Schweizer J (2005). Keratins of the human hair follicle. International Review of Cytology.

[ref-33] Leonti M, Casu L, Raduner S, Cottiglia F, Floris C, Altmann KH, Gertsch J (2010). Falcarinol is a covalent cannabinoid CB1 receptor antagonist and induces pro-allergic effects in skin. Biochemical Pharmacology.

[ref-34] Litvinov IV, Bizet AA, Binamer Y, Jones DA, Sasseville D, Philip A (2011). CD109 release from the cell surface in human keratinocytes regulates TGF-beta receptor expression, TGF-beta signalling and STAT3 activation: relevance to psoriasis. Experimental Dermatology.

[ref-35] Lu Z, Hasse S, Bodo E, Rose C, Funk W, Paus R (2007). Towards the development of a simplified long-term organ culture method for human scalp skin and its appendages under serum-free conditions. Experimental Dermatology.

[ref-36] Maccarrone M, Di Rienzo M, Battista N, Gasperi V, Guerrieri P, Rossi A, Finazzi-Agro A (2003). The endocannabinoid system in human keratinocytes. Evidence that anandamide inhibits epidermal differentiation through CB1 receptor-dependent inhibition of protein kinase C, activation protein-1, and transglutaminase. Journal of Biological Chemistry.

[ref-37] Moll R, Divo M, Langbein L (2008). The human keratins: biology and pathology. Histochemistry and Cell Biology.

[ref-38] Mommers JM, van Rossum MM, van Erp PE, van De Kerkhof PC (2000). Changes in keratin 6 and keratin 10 (co-)expression in lesional and symptomless skin of spreading psoriasis. Dermatology.

[ref-39] Namazi MR (2005). Cannabinoids, loratadine and allopurinol as novel additions to the antipsoriatic ammunition. Journal of the European Academy of Dermatology and Venereology.

[ref-40] Navarro JM, Casatorres J, Jorcano JL (1995). Elements controlling the expression and induction of the skin hyperproliferation-associated keratin K6. Journal of Biological Chemistry.

[ref-41] Paladini RD, Takahashi K, Bravo NS, Coulombe PA (1996). Onset of re-epithelialization after skin injury correlates with a reorganization of keratin filaments in wound edge keratinocytes: defining a potential role for keratin 16. Journal of Cell Biology.

[ref-42] Paradisi A, Pasquariello N, Barcaroli D, Maccarrone M (2008). Anandamide regulates keratinocyte differentiation by inducing DNA methylation in a CB1 receptor-dependent manner. Journal of Biological Chemistry.

[ref-43] Pertwee RG, Howlett AC, Abood ME, Alexander SP, Di Marzo V, Elphick MR, Greasley PJ, Hansen HS, Kunos G, Mackie K, Mechoulam R, Ross RA (2010). International Union of Basic and Clinical Pharmacology. LXXIX. Cannabinoid receptors and their ligands: beyond CB(1) and CB(2). Pharmacological Reviews.

[ref-44] Poeggeler B, Knuever J, Gaspar E, Biro T, Klinger M, Bodo E, Wiesner RJ, Wenzel BE, Paus R (2010). Thyrotropin powers human mitochondria. FASEB Journal.

[ref-45] Pucci M, Pasquariello N, Battista N, Di Tommaso M, Rapino C, Fezza F, Zuccolo M, Jourdain R, Finazzi Agro A, Breton L, Maccarrone M (2012). Endocannabinoids stimulate human melanogenesis via type-1 cannabinoid receptor. Journal of Biological Chemistry.

[ref-46] Radosa J, Dyck W, Goerdt S, Kurzen H (2011). The cholinergic system in guttate psoriasis with special reference to mast cells. Experimental Dermatology.

[ref-47] Ramot Y, Biro T, Tiede S, Toth BI, Langan EA, Sugawara K, Foitzik K, Ingber A, Goffin V, Langbein L, Paus R (2010). Prolactin – a novel neuroendocrine regulator of human keratin expression *in situ*. FASEB Journal.

[ref-48] Ramot Y, Gaspar E, Dendorfer A, Langbein L, Paus R (2009). The ‘melanocyte-keratin’ mystery revisited: neither normal human epidermal nor hair follicle melanocytes express keratin 16 or keratin 6 *in situ*. British Journal of Dermatology.

[ref-49] Ramot Y, Tiede S, Biro T, Abu Bakar MH, Sugawara K, Philpott MP, Harrison W, Pietila M, Paus R (2011). Spermidine promotes human hair growth and is a novel modulator of human epithelial stem cell functions. PLoS ONE.

[ref-50] Richardson JD, Kilo S, Hargreaves KM (1998). Cannabinoids reduce hyperalgesia and inflammation via interaction with peripheral CB1 receptors. Pain.

[ref-51] Roelandt T, Heughebaert C, Bredif S, Giddelo C, Baudouin C, Msika P, Roseeuw D, Uchida Y, Elias PM, Hachem JP (2012). Cannabinoid receptors 1 and 2 oppositely regulate epidermal permeability barrier status and differentiation. Experimental Dermatology.

[ref-52] Ronpirin C, Tencomnao T (2012). Effects of the antipsoriatic drug dithranol on E2A and caspase-9 gene expression in vitro. Genetics and Molecular Research.

[ref-53] Rotty JD, Coulombe PA (2012). A wound-induced keratin inhibits Src activity during keratinocyte migration and tissue repair. Journal of Cell Biology.

[ref-54] Ryle CM, Breitkreutz D, Stark HJ, Leigh IM, Steinert PM, Roop D, Fusenig NE (1989). Density-dependent modulation of synthesis of keratins 1 and 10 in the human keratinocyte line HACAT and in ras-transfected tumorigenic clones. Differentiation.

[ref-55] Saelee C, Thongrakard V, Tencomnao T (2011). Effects of Thai medicinal herb extracts with anti-psoriatic activity on the expression on NF-kappaB signaling biomarkers in HaCaT keratinocytes. Molecules.

[ref-56] Stander S, Reinhardt HW, Luger TA (2006). Topical cannabinoid agonists. An effective new possibility for treating chronic pruritus. Hautarzt.

[ref-57] Stander S, Schmelz M, Metze D, Luger T, Rukwied R (2005). Distribution of cannabinoid receptor 1 (CB1) and 2 (CB2) on sensory nerve fibers and adnexal structures in human skin. Journal of Dermatological Science.

[ref-58] Stenn KS (1981). Epibolin: a protein of human plasma that supports epithelial cell movement. Proceedings of the National Academy of Sciences of the United States of America.

[ref-59] Sugawara K, Biro T, Tsuruta D, Toth BI, Kromminga A, Zakany N, Zimmer A, Funk W, Gibbs BF, Zimmer A, Paus R (2012). Endocannabinoids limit excessive mast cell maturation and activation in human skin. Journal of Allergy and Clinical Immunology.

[ref-60] Suttle MM, Nilsson G, Snellman E, Harvima IT (2012). Experimentally induced psoriatic lesion associates with interleukin (IL)-6 in mast cells and appearance of dermal cells expressing IL-33 and IL-6 receptor. Clinical and Experimental Immunology.

[ref-61] Telek A, Biro T, Bodo E, Toth BI, Borbiro I, Kunos G, Paus R (2007). Inhibition of human hair follicle growth by endo- and exocannabinoids. FASEB Journal.

[ref-62] Thakoersing VS, Danso MO, Mulder A, Gooris G, El Ghalbzouri A, Bouwstra JA (2012). Nature versus nurture: does human skin maintain its stratum corneum lipid properties *in vitro*?. Experimental Dermatology.

[ref-63] Toruniowa B, Jablonska S (1988). Mast cells in the initial stages of psoriasis. Archives for dermatological research. Archives of Dermatological Research.

[ref-64] Toth BI, Dobrosi N, Dajnoki A, Czifra G, Olah A, Szollosi AG, Juhasz I, Sugawara K, Paus R, Biro T (2011). Endocannabinoids modulate human epidermal keratinocyte proliferation and survival via the sequential engagement of cannabinoid receptor-1 and transient receptor potential vanilloid-1. Journal of Investigative Dermatology.

[ref-65] Toth BI, Geczy T, Griger Z, Dozsa A, Seltmann H, Kovacs L, Nagy L, Zouboulis CC, Paus R, Biro T (2009). Transient receptor potential vanilloid-1 signaling as a regulator of human sebocyte biology. Journal of Investigative Dermatology.

[ref-66] Van Dross R, Soliman E, Jha S, Johnson T, Mukhopadhyay S (2012). Receptor-dependent and receptor-independent endocannabinoid signaling: a therapeutic target for regulation of cancer growth. Life Sciences.

[ref-67] Walker JM, Hohmann AG (2005). Cannabinoid mechanisms of pain suppression. Handbook of Experimental Pharmacology.

[ref-68] Weiss RA, Eichner R, Sun TT (1984). Monoclonal antibody analysis of keratin expression in epidermal diseases: a 48- and 56-kdalton keratin as molecular markers for hyperproliferative keratinocytes. Journal of Cell Biology.

[ref-69] Wilkinson JD, Williamson EM (2007). Cannabinoids inhibit human keratinocyte proliferation through a non-CB1/CB2 mechanism and have a potential therapeutic value in the treatment of psoriasis. Journal of Dermatological Science.

[ref-70] Wright K, Rooney N, Feeney M, Tate J, Robertson D, Welham M, Ward S (2005). Differential expression of cannabinoid receptors in the human colon: cannabinoids promote epithelial wound healing. Gastroenterology.

[ref-71] Zhao Y, Gartner U, Smith FJ, McLean WH (2011). Statins downregulate K6a promoter activity: a possible therapeutic avenue for pachyonychia congenita. Journal of Investigative Dermatology.

